# How senior paramedics decide to cease resuscitation in pulseless electrical activity out of hospital cardiac arrest: a mixed methods study

**DOI:** 10.1186/s13049-021-00946-7

**Published:** 2021-09-16

**Authors:** Ali Coppola, Sarah Black, Ruth Endacott

**Affiliations:** 1 MClinRes Research Paramedic, South Western Ambulance Service NHS Foundation Trust, Abbey Court, Eagle Way, Exeter, UK; 2 Head of Research, Audit and Quality Improvement, South Western Ambulance Service NHS Foundation Trust, Exeter, UK; 3 School of Nursing and Midwifery (Faculty of Health: Medicine, Dentistry and Human Sciences), University of Plymouth, Plymouth, UK; 4 School of Nursing and Midwifery, (Faculty of Medicine, Nursing and Health Sciences), Monash University, Melbourne, Australia

**Keywords:** Paramedic, Pulseless electrical activity, Out of hospital cardiac arrest, Resuscitation

## Abstract

**Background:**

Evidenced-based guidelines on when to cease resuscitation for pulseless electrical activity are limited and support for paramedics typically defaults to the senior clinician. Senior clinicians include paramedics employed to work beyond the scope of clinical guidelines as there may be a point at which it is reasonable to cease resuscitation. To support these decisions, one ambulance service has applied a locally derived cessation of resuscitation checklist. This study aimed to describe the patient, clinical and system factors and examine senior clinician experiences when ceasing resuscitation for pulseless electrical activity.

**Design and methods:**

An explanatory sequential mixed method study was conducted in one ambulance service in the South West of England. A consecutive sample of checklist data for adult pulseless electrical activity were retrieved from 1st December 2015 to 31st December 2018. Unexpected results which required exploration were identified and developed into semi-structured interview questions. A purposive sample of senior clinicians who ceased resuscitation and applied the checklist were interviewed. Content framework analysis was applied to the qualitative findings.

**Results:**

Senior clinicians ceased resuscitation for 50 patients in the presence of factors known to optimise survival: Witnessed cardiac arrest (n = 37, 74%), bystander resuscitation (n = 30, 60%), defibrillation (n = 22, 44%), return of spontaneous circulation (n = 8, 16%). Significant association was found between witnessed cardiac arrest and bystander resuscitation (*p* = .00). Six senior clinicians were interviewed, and analysis resulted in four themes: defining resuscitation futility, the impact of ceasing resuscitation, conflicting views and clinical decision tools. In the local context, senior clinicians applied their clinical judgement to balance survivability. Multiple factors were considered as the decision to cease resuscitation was not always clear. Senior clinicians deviated from the checklist when the patient was perceived as non-survivable.

**Conclusion:**

Senior clinicians applied clinical judgement to assess patients as non-survivable or when continued resuscitation was considered harmful with no patient benefit. Senior clinicians perceived pre-existing factors with duration of resuscitation and clinical factors known to optimise patient survival. Future practice could look beyond a set criteria in which to cease resuscitation, however, it would be helpful to investigate the value or threshold of factors associated with patient outcome.

## Background

In the United Kingdom (UK), pulseless electrical activity (PEA) is the first documented rhythm in 32.7% of adult out of hospital cardiac arrests (OHCA), with 5.3% of patients surviving to hospital discharge [1]. The incidence of PEA has increased year on year from 21.17% in 2014 with little improvement to patient outcomes. UK paramedics manage PEA according to advanced life support (ALS) guidelines provided by the Joint Royal College Ambulance Liaison Committee (JRCALC), informed by the UK and European Resuscitation Councils [2]. JRCALC guidelines state there is limited evidence on when to start, continue or cease resuscitation. It is well acknowledged that research focusing on PEA is particularly challenging [3]. Limited diagnostic capabilities and treatment options outside that of standard ALS further limit the evidence base. However, early recognition of cardiac arrest, presence of basic cardiopulmonary resuscitation, defibrillation and post resuscitation care can optimise patient survival [4]. In contrast, long duration of resuscitation, morbidity, absent return of spontaneous circulation (ROSC) and reversible causes are factors found in patients unlikely to survive [2].

Unwitnessed cardiac arrest, no basic life support and a persistent non-shockable cardiac arrest rhythm was previously applied as termination of resuscitation criteria. However, research to validate this criteria found unexpected survivors (9%). Therefore, no termination criteria have been adopted into clinical practice [5]. Clinical guidelines in the UK suggest paramedics request senior clinician advice as PEA presents a challenge to decision-making and for some patients there may be a point where it is reasonable to cease resuscitation [2].

OHCA were previously found to be resuscitated in non-survivable circumstances [6]. To continue resuscitation in patients with no hope of survival raises an ethical debate regarding patient care and dignity in death [7]. The European Resuscitation Council congress 2021 highlighted the importance of resuscitation decisions from an ethical perspective, considering context of best interest decisions and where the harm versus the benefit of resuscitation are fully appreciated by the clinician. Certainly, approaches to the ethics of resuscitation vary, however, in the UK paramedics work within their scope of clinical practice and boundaries of employment [8], with limited autonomy to implement beneficence and non-maleficence to withhold resuscitation without senior clinician involvement.

In one UK ambulance service, a team of senior clinicians, termed senior clinical advisors (SCAs) are autonomous paramedics who work outside the usual scope of paramedic practice [9]. SCAs provide 24 h ‘on call’ remote telephone support to on-scene paramedics who lack the clinical autonomy to cease resuscitation for adult PEA. SCAs apply a locally derived checklist amended from JRCALC guidelines [2] to support and document cessation of resuscitation. To our knowledge how SCAs make the decision to cease resuscitation has not previously been explored. In this study we aim to describe the patient, clinical and system factors of PEA and examine SCA experiences when ceasing resuscitation.

### Study objectives

1. To describe patient, clinical and system factors of out of hospital cardiac arrest patients with PEA when resuscitation was ceased by an SCA.
2. To examine associations between variables known to optimise patient survival.
3. To explore the experiences of SCAs making cessation of resuscitation decisions.

### Patient and public involvement and engagement (PPIE)

PPIE to prioritise research in the public interest and improve the quality of research is an expected commitment when conducting research in the UK [10]. This study was presented to the participating ambulance service PPIE group. The group consisted of eight members including one cardiac arrest survivor and five research paramedics. The consensus was an appropriate research question and methodology. The group highlighted paramedics should cease resuscitation based on individual circumstances, with family wishes and quality of life important factors to consider when collecting data. The group highlighted that not all paramedics would seek SCA advice and the sample size may be small. Interviews were felt appropriate to collect qualitative data given the sensitive nature of resuscitation decisions. However, it was felt a range of interview platforms should be offered, face to face, video, telephone or email due to the geographical location and shift work commitments of participants.

### Design and methods

#### Mixed methods design

In emergency care mixed methodology provides a pragmatic approach to integrate both qualitative and quantitative paradigms resulting in a better understanding of the research findings compared to one method alone [11]. The objectives of this study required both quantitative and qualitative methods and a pragmatic epistemology offered the opportunity to gain new knowledge by embracing both [12]. The mixed methods two-phase explanatory sequential design was selected most appropriate to answer the research question. Quantitative data was collected and analysed followed by qualitative data to help explain the connected points found in the results (Table 1) [12].

**Table 1:** Explanatory sequential design

Quantitative study	Describe the adult patient, clinical and system factors of pulseless electrical activity
Quantitative results	Identify the quantitative results which require further explanation using a qualitative methodology
Qualitative study	Apply the quantitative results to inform the semi-structured interview questions
Summarise and interpret results	What are connective points between the qualitative findings help to explain the quantitative results?

#### Quantitative data collection

Cessation of resuscitation checklists for PEA were routinely collected and the primary source of the quantitative data (“Appendix 1”). The checklists recorded patient demographics, clinical findings and a free text box to document the rationale for ceasing the resuscitation. A retrospective consecutive sample was collected from December 2015 to December 2018. Additional data items were sourced from patient clinical records, computer aided dispatch system and the cardiac arrest registry comprising of patient, system and clinical factors (Table 2).

**Table 2:** Variables for analysis

Patient factors	System factors	Clinical factors
Age	Time of collapse	Adrenaline dose (mg)
Gender	Time to basic resuscitation	Progression to a shockable rhythm
Arrest witnessed	Time to advanced resuscitation	Defibrillation
Bystander resuscitation	Duration of arrest	End-tidal carbon dioxide
Co-morbidities	Duration of basic life support (BLS)	Heart rate
Place of arrest	Duration of advanced life support (ALS)	Return of spontaneous circulation
Family wishes considered	Total duration of out of hospital cardiac arrest	Rationale for cessation of resuscitation

Patient identifiable information was redacted, and retrospective consent was not required as data was anonymised.

#### Exclusion criteria

Cases subject to coronial or police investigation and paediatric patients below 18 years were excluded.

#### Quantitative data analysis

Descriptive statistics were applied to all variables. Continuous variables were reported as mean and standard deviation (Sd) for parametric data or median and interquartile range (IQR) for non-parametric data. Categorical variables were summarised as counts and percentages. Free text box information (family wishes, rationale for cessation of resuscitation) was manually grouped into categories of similar meaning and counted. Chi Square and independent t-tests were performed on factors known to optimise survival. *P* values less than 0.05 were considered statistically significant. No power calculation was required for this study. Statistical analysis was conducted using SPSS version 24 [13].

Quantitative data were interpreted for significant and non-significant results, outliers and group differences. Unexpected results were identified when data was not consistent with typical findings of PEA undergoing resuscitation. These results required further explanation and were interpreted to construct the interview questions for the qualitative study [12].

#### Qualitative data collection

A purposive sample of SCAs were selected to provide expert participants who cease resuscitation and complete the checklists. A sample of four to six participants was felt suitable to provide an in-depth examination of the quantitative results [12]. Participants were identified by the ambulance service research team, emailed for expressions of interest and provided with a participant information sheet and research study consent document. A range of interview platforms were offered to reduce barriers of participation. Virtual interviews whilst considered a poor substitute compared to face-to-face interviews offer participants the opportunity to answer questions in their own time, reflect and edit responses, comfort when disclosing sensitive information and validation of responses by following up answers [14].

Semi-structured interview questions were used to collect data whilst enabling participants the freedom to elaborate and explore emerging topics. Eight interview questions were developed and piloted by a critical care paramedic (Table 7). Interviews were conducted by the lead author. Interviews applied reflective validation as an alternative to member checking to reduce bias and increase the trustworthiness and credibility of answers [15]. Confidentiality and anonymity were maintained by allocating non-identifiable codes to each interview. Email correspondence used encryption over a secure NHS network.

#### Qualitative data analysis

Interview transcripts were analysed using content framework analysis. Analysis consisted of five stages (1) familiarisation (2) identifying thematic framework (3) indexing of codes (4) charting of themes (5) mapping and interpretation of findings [16]. As interview questions were constructed using the quantitative results, there were some ‘a priori’ themes to guide the initial deductive analysis. Additional themes emerged through inductive analysis. Data coding was managed using NVIVO 11.4.2 [17]. Reflexivity was supported using a diary to bracket assumptions and minimise influence during data collection and analysis. Data was cross coded and verified by two independent researchers of different professional backgrounds. The consolidated criteria for reporting qualitative research checklist (COREQ) was applied [12].

#### Setting

Emergency medical systems in the UK consist of ambulance services, staffed by paramedics trained in ALS. Paramedics are defined as “a registered health professional who work across a range of health and care settings, as well as in education, leadership and research” [9]. This research study was conducted in a single ambulance service operating 100 sites across an area of 10,000 mi^2^ with a population of 5.5 million. The ambulance service attends up to 3000 incidents per day and approximately 4000 resuscitations each year [18].

#### Ethics

Institutional ethics approval was provided by the ambulance service (ref 17-018) and the University (reference 1718424).

## Results

Cardiac arrest registry data was available for 32 months from 1st April 2016 to 31st December 2018. The total number of cardiac arrests were 10,246 and 1823 reported PEA as the initial rhythm. The average annual cardiac arrest rate was 3842. The annual PEA rate was 683: the incidence of PEA was 18% of these 54% (n = 368) of patients were transported to hospital; 46% (n = 315) of patients died at scene and 6% (n = 43) of patients survived to hospital discharge. During the study period SCAs were contacted regarding 57 patients. SCAs requested paramedics continue resuscitation for seven patients (12%) and resuscitation was ceased in 50 patients (88%). The patient, system and clinical factors of the 50 patients where resuscitation was ceased were analysed in detail. As a yearly average 6% (n = 19) of PEA patients who died at the scene of the cardiac arrest had SCA involvement.

### Patient factors

In one ambulance service SCAs ceased resuscitation for 50 patients with PEA. The median age was 78.50 years (*IQR*: 69, 84). The majority of patients were male (n = 30, 60%), located at a residential address (n = 44, 88%), had a witnessed cardiac arrest (n = 37, 74%) and received basic resuscitation (n = 30, 60%). Co-morbidities were documented for the majority of patients (n = 40, 80%) of which 51% were cardiac with a previous history of myocardial infarction, aortic aneurysm, cardiac failure, hypertension and 8% respiratory with chronic obstructive pulmonary disease. Family wishes were documented in the free text box, cross referenced with the patient clinical record and considered in 32% (n = 16) of patients.

### System factors

System factors are reported in Table 3. The duration of BLS and lone ambulance BLS were similar (0:07:30, 0:07:35). Duration of ALS was below 55 min, however, the total duration of OHCA was nearly 1 h and 10 min when no flow and BLS times were included.

**Table 3:** System factors

System factors	N (%)	Duration hr:min:sec
No flow time^&^ *median (IQR)*	50 (100)	0:07:30 (4.46–15.25)
Duration of BLS *median (IQR)*	26 (52)	0:07:35 (4.58–16.01)
Duration of lone ambulance BLS^€^ *median (IQR*)	23 (46)	0:07:32 (5.31–15.9)
Duration of ALS^#^ *mean (SD)*	50 (100)	0:54:46 (0:16:59)
Duration of resuscitation^^^ (BLS & ALS) *mean (SD)*	50 (100)	0:59:32 (0:17:39)
Total duration of out of hospital cardiac arrest *mean (SD)*	50 (100)	1:09:23 (0:19:53)

^&^No flow time, time from collapse to the start of resuscitation

^€^Lone ambulance responders (community responders, advanced technicians, paramedics, duty officers) providing BLS only

^#^ALS time of ambulance arrival to confirmation of death

^^^Duration of out of hospital cardiac arrest, no flow time to confirmation of death

### Clinical factors

Clinical factors are reported in Table 4. PEA was the only reported rhythm for 18 patients (36%). A rhythm change was reported in 32 patients (64%), 12 patients (26%) intermittently transitioned to asystole and 20 patients (38%) converted to a shockable rhythm. ROSC was achieved intermittently and was not sustained for longer than 10 min. ETCO_2_ value was recorded prior to confirmation of death. A median dose of 6 mg of adrenaline was administered. Guidelines state 1 mg every 3–5 min, however, this study reports a mean ALS duration of 54 min. This duration should have resulted in a cumulative adrenaline dose of 11 mg. This may reflect the time to venous access or interruptions caused by rhythm changes, defibrillation or ROSC.

**Table 4:** Clinical factors

Clinical factors	N (%)	
Defibrillation	22 (44)	
Return of spontaneous circulation (ROSC)	8 (16)	
End-tidal carbon dioxide (ETCO_2_) (kilopascal, kPa) (Mean)	44 (88)^a^	2.3 (2.0–4.4)
Adrenaline Dose (1 mg/10 ml) *median (IQR*)	50 (100)	6.0 (5.0–7.5)
PEA rate < 50 beats per minute (BPM)	46 (92)	

^a^Missing ETCO_2_ data for 6 patients

The association between factors known to optimise patient survival are reported in Tables 5 and 6. The only significant association was found between witnessed cardiac arrest and bystander resuscitation (χ^2^ *n* = 50, *df* 1 = *p* 0.000).

**Table 5:** Association between factors known to optimise patient survival

Factors		Result
Witnessed cardiac arrest	Bystander BLS	*p* 0.000
Witnessed cardiac arrest	Defibrillation	*p* 0.856
Bystander BLS	Defibrillation	*p* 0.103
Witnessed cardiac arrest	ROSC	*p* 0.418
Bystander BLS	ROSC	*p* 0.875

**Table 6:** Duration of ALS for patients with ROSC/no ROSC and defibrillation/no defibrillation

	No defib *M*(SD)	Defib *M*(SD)	*p*-value	No ROSC *M*(SD)	ROSC *M*(SD)	*p*-value
Duration of ALS^$^	53:25 (0:16.45)	56.29 (0:17.31)	0.531	53:32 (0:17.34)	1:01:16 (0:12.25)	0.242

^$^T-test

### SCA rationale for ceasing resuscitation

In the checklist free text box, SCAs documented 161 reasons for ceasing resuscitation. Reasons were grouped into similar meaning, counted and categorised. Clinical factors were most considered, low ETCO_2_ values, no ROSC and persistent PEA (n = 117, 71.7%). Patient factors included family wishes and progressive illness (n = 30, 19.6%). System factors included the duration of resuscitation and extended transfer times to hospital (n = 14, 8.5%).

The results identified a number of PEA patients presenting with factors known to optimise survival; witnessed cardiac arrest, bystander resuscitation, defibrillation, ETCO_2_ value and ROSC, albeit intermittent. These results were used to construct the semi-structured interview questions to examine in detail why the resuscitation was ceased (Table 7).

**Table 7:** Results for further examination

Quantitative result	Question
Witnessed cardiac arrest 74%	1. How do witnessed cardiac arrest influence a cessation decision?
Bystander BLS 60%	2. How does bystander resuscitation influence a cessation decision?
Defibrillation 44%	3. How do rhythm changes influence a cessation decision?
Mean end-tidal carbon dioxide value 2.3 kPa	4. What are your views on end-tidal values when ceasing resuscitation?
Mean duration of resuscitation 54 min	5. Is there a duration of resuscitation beyond which you would cease resuscitation?
Multiple factors reported when ceasing resuscitation	6. Which factors influence your resuscitation decisions the most?
Variation in rationales to cease resuscitation	7. Why was there variation in the rationales to cease resuscitation?
SCA involvement in 6% of patients who died at the scene	8. What are your thoughts on SCA support and remote decisions?

### Interview findings

Six SCAs participated. Five telephone and one email interview were conducted. SCAs were mixed gender, the average age was 40 years (35–47 years), clinical experienced averaged 14 years and ‘on call’ rota experience ranged between 6 months and 9 years. Telephone interviews lasted between 28–54 min. The email interview dialogue was developed over several days to provide an in-depth narrative of answers.

Data analysis generated 349 codes, 12 sub-themes and four main themes. One ‘a priori’ theme was derived from the quantitative data: *defining a futile resuscitation*. Three further themes emerged during data analysis, *the impact of ceasing resuscitation*, *perceived conflict between the SCA and on-scene paramedics* and *supportive tools for ceasing resuscitation*. Illustrative data excerpts are presented in Table 8.

**Table 8:** Illustrative data excerpts

Theme	Defining a futile resuscitation
Key features	*“things like age, rhythm, end tidal capnography, previous medical conditions and events preceding the event are probably some of the key factors I’d be thinking about.” (SP1)*
A poor quality of life	*“quality of life I know there’s lots of controversy about should you use that as an indicator, but I find it useful to know in terms of if in the nicest possible way if the patients in a nursing home bedbound with severe dementia and comorbidities there’s something about should we be resuscing anyway.” (SP5)*
A perceived natural end of life	*“I may feel more confident to say actually if it was an older person or more of the elder category then to consider that that might be their natural end of life.” (SP3)*

Theme	The impact of ceasing resuscitation
Confidence gained with experience and exposure	*“I am confident in making a decision yes I’ve been making it for enough years so I feel confident in making it…. is it always clear cut? No.” (SP5)*
Critical awareness and self -reflection	*“I feel confident with my decisions but I do rethink every decision that I made remotely” (SP3)*
The weight of responsibility when deciding futility	*“It did have its moments going about your daily business and you think bloody hell that is a bit hardcore so it’s very easy writing out a word document on your form and filling it in but when you actually put someone’s name in it and all their bits and pieces its quite oh actually someone just died and sometimes that was more stressful more poignant that being there.” (SP4)*

Theme	Perceived conflict between senior and on-scene paramedics
Senior paramedic/SCA? clinical knowledge, experience and accountability	*I don’t think crews have a great understanding of PEA they don’t necessarily know or fully understand the potential for reversible causes”. (SP2)*
Clinical leadership and moral judgements	*“I would say that from my experience of it that you can make a decision with a degree of separation from what’s going on so you can take a reasoned history and then consider the factors without the background noise if you like and the human factors that come into play from a stressful on scene environment”. (SP4)*
Conflict between senior and on-scene paramedic	*“They are trying to sell me a situation to fulfil their own agenda so their selling me a patient that’s profoundly unwell when there actually a survivable aspect to it because it is frustrating”. (SP4)*

Theme	Supportive tools for cessation of resuscitation decisions
The ‘checklist’ as a safety net	*“I think the fallback option which is used is just follow a checklist in order for safe practice.” (SP1)*
Checklist deviation and sound clinical reasoning	*“Cessation of resuscitation checklist which we should use which helps govern our decision making……if a patient doesn’t fall within that then we have to be pretty confident and be very careful about calling a cessation of resuscitation attempt.” (SP1)*
Checklist and moving forward	*“I do think the checklist that we are using is almost double negative in terms of the questioning and it’s still a little ambiguous in some areas.” (SP3)*

### Defining a futile resuscitation

SCAs applied multiple factors to identify when ceasing resuscitation was appropriate. No one factor was considered, however, age, co-morbidity, persistent PEA, morphology, low ETCO_2,_ no or intermittent ROSC and prolonged resuscitation were highlighted along with a perceived poor quality of life or a natural end of life event.

I think about that question in context things like age, rhythm, end tidal capnography, heart rate, morphology previous medical conditions and events preceding the event are probably some of the key factors I’d be thinking about. (SP1)

SCAs described interpreting these factors to provide a good history of the event and a holistic view of the patient. SCAs then applied their knowledge, experience and acumen to determine if the patient had reached a natural end of life or was beyond surviving.

An older person I’m more likely to allow them to cease a PEA but that would be based on what their quality of life is like, if they are in a nursing home bedbound or they play golf and run marathons its taking their quality of life as one of the considerations. (SP2)

### The impact of ceasing resuscitation

Repeated exposure to ceasing resuscitation increased confidence and the decision-making process became more comfortable over time; however, SCAs highlighted that the decision to cease resuscitation was not always easy or clear.

I got to a position of confidence, sometimes I had to work at, it I never felt it was a wrong decision to make or that I was second guessing myself once I’ve hung up but on occasion it took me a while to get there. (SP4)

SCAs carefully considered and reflected upon their decisions. Despite being relatively confident ceasing the resuscitation was correct, the decision could carry an emotional burden. Resuscitation decisions when woken at night were found particularly challenging with human factors adding to the complexity of decision-making.

They are difficult decisions to make and are often disruptive, especially at night when you are woken up, often I find it difficult to fall back to sleep as I reflect on my decision and feel a sense of loss for the family. (SCA6)

### Perceived conflict between the SCA and on-scene paramedic

A number of SCAs highlighted conflict between themselves and the on-scene paramedic. SCAs felt pressured to cease resuscitation and found paramedics were not always prepared to discuss the survivable aspects.

I think paramedics maybe on the whole need a bit more education around PEA and when patients are survivable. (SP1)

SCAs felt remote telephone clinical support provided a degree of separation, enabling the time required to consider all factors to make an informed decision. SCAs acknowledged the limitations of remote support and the disadvantages of not visually interpreting the scene of the OHCA. However, SCAs felt their clinical knowledge and experience informed the decision-making process to ensure decisions were made in the best interest of the patient. At times SCAs felt paramedics lacked the clinical knowledge and were not comfortable with the responsibility and accountability required to cease resuscitation.

Sometimes they are wanting someone else to make that decision, they can say the SCA allowed them to cease resuscitation so I think some paramedics are like that, that someone else has made the decision and I am off the hook, yet we are all registered professionals all accountable. (SP2)

### Supportive tools for ceasing resuscitation

SCAs referred to the PEA cessation of resuscitation checklist and felt it worked well as a supportive decision tool. The checklist was positively viewed with some uncertainty on how to better standardise the complexities of resuscitation decisions.

Deviation from the checklist was possible with careful considered clinical judgement.

we’ve got the checklist we can still step outside the check list… but I think if you’ve got all the SCAs in a room and ran some scenarios I’m not entirely convinced they’d make the same consistent decision. (SP5)

This occurred when resuscitation was ceased in patients with factors known to optimise survival; witnessed collapse, bystander resuscitation, ROSC or defibrillation. This deviation may have involved one or all of the factors, introducing variation in the care delivered to patients with one SCA stating inconsistent decision-making in how the checklist was applied.

I do think the checklist is almost double negative in terms of the questioning and it’s still is a little ambiguous, though a more structured form would lead to making the decision to not cease or convey more PEAs, but I do think it probably is a bit dependent on the individual that takes the call to the level of the decision. (SP3)

### Synthesis of findings

The connective points between the quantitative results and qualitative findings were summarised into three categories (1) PEA within a local context (2) SCA experiences of ceasing resuscitation (3) multifactorial decision-making. This process is presented in “Appendix 2”.

### PEA within a local context

The results found 46% of PEA patients died at the scene of the cardiac arrest, however, only 6% of these had SCA involvement. One explanation may be the conflict perceived by the SCA and the on-scene paramedic. The SCAs felt the on-scene paramedic did not want to discuss the survivable aspects of the cardiac arrest and were telephoning to cease the resuscitation as this was outside their scope of practice.

### Senior clinical advisor experiences of ceasing resuscitation

SCAs documented 161 reasons for ceasing the resuscitation in the free text box of the checklist. Factors known to optimise patient survival were present, however, SCAs felt continued resuscitation was not in the best interest of the patient. SCAs felt these patients were not survivable due to a perceived poor quality of life, informed by frailty or morbidity. They felt ceasing resuscitation was not always easy or clear and relied on balancing multiple factors to make a best interest decision.

### Multifactorial decision-making

SCA decision-making was mostly informed by clinical factors. SCAs had different perceptions on the value or threshold of factors which made continued resuscitation inappropriate. One SCA said resuscitation was futile after 20 min, another 45 min and another 1 h. That’s said, decision making was not based on one factor alone and not all factors such as quality of life were easily measurable with different perceptions. SCA decision-making applied clinical judgement informed by their knowledge, experience and acumen.

## Discussion

This study examined how SCAs ceased resuscitation in patients with PEA. Factors known to optimise survival were present within the sample of patients, witnessed cardiac arrest, bystander BLS, defibrillation and ROSC, as evidenced in the chain of survival [19]. Witnessed cardiac arrest and bystander BLS were statistically significant, a similar result to that found in other studies [20]. In this study, defibrillation was administered following conversion from PEA to a shockable rhythm (n = 22, 44%). In a previous study conversion was associated with ROSC but not survival to hospital discharge [21]. SCAs viewed conversion and defibrillation as a positive progression, however, patients who remained in PEA prior to ROSC were previously found to have a more favourable neurological outcome compared to those who converted [22]. ROSC was also viewed positively by the SCAs, however, in this study ROSC was achieved intermittently and not sustained (n = 8, 16%).

Electrocardiogram (ECG) morphology was well considered with the majority of patients reporting a heart rate below 50 bpm (n = 46, 92%). A slow heart rate is widely accepted as a poor prognostic marker for survival [23], however, one study found morphology inaccurate to predict patient outcome [24]. A slow wide morphology indicates no cardiac movement, often categorised as a true PEA. A fast narrow morphology, described as pseudo PEA may present with profound hypotension and some cardiac movement [25]. However, there are no clear predefined categories of PEA with one study applying six different morphologies, albeit none associated with survival [26]. The SCAs considered a fast narrow morphology more survivable and this view was supported by one study which found pseudo PEA often mistaken for ROSC. ECG, ETCO_2_ and ultrasound collectively can help identify ROSC, however, ultrasound is not routinely used by paramedics [27]. ETCO_2_ is routinely monitored and in this study the mean was 2.3 kPa. A systematic review investigated ETCO_2_ to prognosticate resuscitation futility and whilst associations were found, no specific value to cease resuscitation could be determined [28]. SCAs reported no optimal value or threshold to cease resuscitation and this was reflected in the literature, suggesting a further investigation to associate ETCO_2_ and morphology with outcome is required. SCAs considered duration of resuscitation the most important system factor. Once again, previous research was unable to identify an optimal duration with studies reporting anywhere between 10 to 47 min [19, 29]. In this study, the mean ALS resuscitation (54 min) was considered non-survivable by the majority of SCAs.

Patient factors advanced age, frailty and morbidity were interpreted to help the SCAs understand the patients quality of life to inform the possibility of a natural end of life event. SCAs recognised variation in the perceptions surrounding quality of life and previously no accepted method of assessment were identified as an accurate measure [30]. Nor was age found to be an accurate measure of patient outcome [31, 22, 23]. Previous data on pre-hospital clinical frailty and morbidity and the impact on survival was limited, however, these factors were found to inform resuscitation decisions [29]. In these circumstances, cardiac arrest was not reversible, SCAs were confident to cease resuscitation and were well supported by the guidelines to do so [32]. SCAs acknowledged their perceptions introduced variation in the care delivered to patients, even when applying the checklist to support decision-making.

In this study, SCAs viewed the checklist as a supportive tool, to help reduce human factors. This view was supported by previous studies which found decision tools for cardiac arrest likely to identify patient survival and reduce variation in practice [33, 34]. That said, another study found checklists to support clinical decisions insufficient as they omitted the multiple factors required to build a holistic view of the patient [35]. This view reflected SCA practice as deviation from the checklist occured when clinical judgement balanced the factors as non-survivable, however, making the decision to cease resuscitation presented a number of challenges.

The SCAs experienced conflict with the paramedics on-scene, which they felt was due to a lack of training or knowledge and an unwillingness to discuss the survivable aspects. This finding was similar to that of a previous study which found conflict between clinicians due to expectation, pressure, personal beliefs and moral practice [36]. Previously paramedics reported feeling underconfident when making resuscitation decisions [37]. SCA forms part of the clinical guideline which underpins UK paramedic practice [2]. In this study SCAs felt shared decision making was beneficial, providing time for reflection to reduce human factors, although the effectiveness of remote support in emergency care has not previously been assessed [38, 39]. Also, from a legal perspective, uncertainty was found regarding which clinician was liable for the decisions, the on-scene clinician or remote support [40]. Interestingly in this study, paramedics made the majority of resuscitation decisions as SCA involvement was limited. Given the paucity of evidence-based guidelines, this finding requires further exploration.

## Limitations

The main limitation of this study was the small sample size. No power calculation was applied as there were no predefined criteria for ceasing resuscitation for PEA. This may have introduced a type II error. Consecutive sampling was applied to capture all SCA checklist data between December 2015–2018. This data was screen by two reviewers against the inclusion criteria, however, the sample reflects the limited involvement of SCAs. Missing data was identified for ETCO_2_. Severity of morbidity was not reported, nor were clinical frailty scores. The effectiveness of bystander resuscitation or by whom it was conducted was not measured. Researcher bias was acknowledged when conducting the interviews. To reduce bias questions were piloted and data was cross coded by two independent reviewers of different professional backgrounds. One interview was conducted by email, limiting the opportunity to probe the participant responses.

## Conclusion

SCAs applied clinical judgement to assess patients as non-survivable or when continued resuscitation was considered harmful with no patient benefit. SCAs perceived pre-existing factors of morbidity, frailty, age and quality of life with duration of resuscitation and the clinical factors known to optimise patient survival. This multifactorial approach questions the appropriateness of ceasing resuscitation based on clinical factors alone. Future practice could look beyond a set criteria in which to cease resuscitation, however, it would be helpful to investigate the value or threshold of factors associated with patient outcome.

## Data Availability

An anonymised patient data set and anonymised participant transcripts are available from the author on request.

## Appendices

### Appendix 2: Framework of results

**Table:**

Quantitative result	Witnessed cardiac arrest 74%	Bystander BLS 60%	Defibrillation 44%	Mean end-tidal carbon dioxide value 2.3 kPa	Mean duration of resuscitation 54 min	Multiple factors reported when ceasing resuscitation	Variation in the rationales to cease resuscitation	SCA involvement in 6% of patients who died at the scene
Qualitative questions	1. How do witnessed cardiac arrest influence a cessation decision?	2. How does bystander resuscitation influence a cessation decision?	3. How do rhythm changes influence a cessation decision?	4. What are your views on end-tidal values when ceasing resuscitation?	5. Is there a duration of resuscitation beyond which you would cease resuscitation?	6. Which factors influence your resuscitation decisions the most?	7. Why was there variation in the rationales to cease resuscitation?	8. What are your thoughts on SCA support and remote decisions?
Qualitative findings	*"Witnessed is more likely to be known, a longer period and obviously the worst outcome." (SCA2)*	“It does make impact on a decision to cease, but there is no way of knowing if that was effective.” (SCA2)	“A rhythm change to asystole is a poor, but any other rhythm changes is a positive.” (SCA1)	“If they’ve been reasonable and deteriorated then again, I’m going to want the whole bigger picture.” (SCA3)	“I think it depends the different factors, anything over 45 min I’m a bit cenacle about.” (SCA5)	“The rate of the PEA the morphology is really relevant.” (SCA1)	“It’s a difficult subjective in hindsight it’s easy when you are look at the resus unfolding in front of you.” (SCA5)	“They are difficult I reflect on my decision and feel a sense of loss for the family.” (SCA6)
Qualitative themes	Defining a futile resuscitation					The impact of ceasing resuscitation	Supportive tools for cessation of resuscitation decisions	Perceived conflict between the senior and on-scene paramedic
Summary and interpretation of results						PEA in a local context		
		SCA experiences of ceasing resuscitation						
	Multifactorial decision-making							

## Abbreviations

ALS: Advanced life support
BLS: Basic life support
ECG: Electrocardiogram
ETCO_2_: End tidal carbon dioxide
PEA: Pulseless electrical activity
ROSC: Return of spontaneous circulation
SCA: Senior clinical advisor
